# Subjective Cognitive Complaints in Schizophrenia: Relation to Antipsychotic Medication Dose, Actual Cognitive Performance, Insight and Symptoms

**DOI:** 10.1371/journal.pone.0083774

**Published:** 2013-12-20

**Authors:** William Sellwood, Anthony P. Morrison, Rosie Beck, Suzanne Heffernan, Heather Law, Richard P. Bentall

**Affiliations:** 1 Institute of Psychology, Health and Society, University of Liverpool, Liverpool, United Kingdom; 2 School of Psychological Sciences, University of Manchester, Manchester, United Kingdom; 3 Division of Health Research, Lancaster University, Lancaster, United Kingdom; Baylor College of Medicine, United States of America

## Abstract

**Background:**

Subjective cognitive complaints are prevalent in those affected by functional psychoses and a variety of possible associated factors have been investigated. However, few studies have examined these potential factors within single studies or analyses.

**Methods:**

Patients with a history of a schizophrenia spectrum disorder (n = 115) and a non-clinical comparison group (n = 45) completed the Subjective Scale to Investigate Cognition in Schizophrenia (SSTICS) and the Brief Assessment of Cognition in Schizophrenia (BACS). The patient group also completed the Positive and Negative Syndromes Scale (PANSS), the Birchwood Insight Scale (IS), and the Hospital Anxiety and Depression Scale (HADS).

**Results:**

The BACS and SSTICS scores were associated in the non-clinical comparison group, but not in the patient group. In the patient group worse subjective cognition was associated positively with good insight, greater dysphoria and greater positive symptoms. Linear regression revealed that, once other variables had been accounted for, dysphoria (HADS anxiety and depression factor) was the only significant predictor of SSTICS scores.

**Conclusions:**

Subjective cognitive impairment in patients with psychosis in the absence of formal testing should not be taken as evidence of impaired cognitive functioning. Mood should be investigated when patients present with subjective cognitive complaints.

## Introduction

Self-report of cognitive deficits in schizophrenia is potentially important. If patients can accurately report their own cognitive problems this should help in the planning of interventions related to functional outcome and individual therapy. In addition, it has been suggested that for some, subjective cognitive complaints (SCC) underlie a wide range of symptoms elicited from patients that develop later as psychosis progresses and that there is therefore an association between SCC and future psychotic symptoms [1]. However, in a number of studies SCC and actual cognitive performance is not associated and in those where there is such an association, the relationship between these two variables is weak[2], [3], [4], [5]. Moreover, specific complaints are not mirrored on relevant specific tests of neuropsychological function. This has been explained in terms of the lack of ecological validity of neuropsychological tests and patients misinterpreting terms such as “attention” and “memory”. This raises the question as to what SCC actually represents and what factors are involved in its expression. A number of factors have been investigated in this regard. Medication side effects [6], [7], [8], insight [4] and symptoms associated with psychosis [9], including depression[2], [10] have all been found to be related to SCC. However, no studies have looked at the relative predictive values of these variables, or whether they contribute to predicting SCC independently or not. The aim of the present study was to replicate the finding that patients' subjective experiences of cognitive functioning fail to reflect objectively assessed cognitive performance[2], [3], [4], [5] and to determine the relative importance of possible predictors of subjective cognitive complaints in schizophrenia. Specifically, once neuropsychological performance and antipsychotic dose had been accounted for, would SCC be predicted in terms of symptoms related directly to psychosis, including insight? In addition, would anxiety and depression predict SCC over and above psychotic symptoms?

## Methods

### Ethical Statement

This study was conducted in accordance with guidelines set by the UK NHS national ethics procedures (National Research Ethics Service - NRES) and approved by the NHS trusts at local levels (reference numbers 09/H1012/9 for clinical group and 10/H1013/5 for comparison group). All participants (including those aged 16 to 17 years) provided written informed consent and were deemed to have capacity to provide informed consent by the clinical team working with them. Recruitment and consent procedures for all participants including those aged 16 to 17 years were approved by the NRES committees.

### Clinical participants

115 patients, 84 male and 31 female, mean age = 36.0 years (SD = 11.59), who had been diagnosed by their consultant psychiatrists as suffering from a schizophrenia-spectrum disorder according to ICD-10 criteria, aged 16–65, with a sufficient level of English literacy to complete the measures and capacity to provide informed consent, were referred from clinical teams in the North West of England. The selection criteria were purposefully broad, in order to recruit patients varying in their symptoms and duration of illness. Although those with acquired brain injury or learning disability were not excluded and presence of such a diagnosis noted, none were recruited into the sample. The majority were White British (83%). Twenty-six had no school qualifications, 35 had completed school level qualifications, 15 had experienced higher education, and the rest had obtained vocational qualifications (data missing from 3). Four were in full or part-time employment and 6 were students. Nine were in long-term relationships and 10 were divorced or separated. Diagnoses, confirmed using ICD-10 checklists, were as follows: schizophrenia (n = 75), schizoaffective disorder (n = 10), unspecified non-organic psychosis (n = 14), acute and transient psychotic disorder (n = 12), and persistent delusional disorder (n = 4). Data on medication were available for 93 patients; of these 11 were not taking antipsychotics and 82 were (mean daily dose = 351.41 mg, SD = 221.04, CPZ equivalent calculated using the method of [11] when possible, otherwise from the British National Formulary (Joint Formulary Committee, 2007). Duration of illness was estimated from participant's reports of age of first psychotic episode and was highly skewed (median = 140 months, range 8–522; data missing from 5 participants). Data for this latter variable were therefore log transformed for use in the analyses reported below.

### Comparison group

The comparison group comprised a convenience sample recruited from local fire services; staff working in National Health Services via posters displayed in office areas; and acquaintances of the research assistants, using a snowballing method. The comparison participants completed the study in the same way as the patients, directly with the researcher, and were remunerated for their time.

Thirty were male and 15 female, with a mean age of 34.6 years (SD = 11.72). Seventeen had obtained school-level qualifications, 17 had experienced higher education and the rest had vocational qualifications. Fifteen were in long-term relationships and 2 were separated.

### Measures

#### Positive and Negative Syndromes Scale (PANSS)

The PANSS [12] is a clinician administered thirty-item semi-structured interview consisting of seven items assessing positive symptoms (*Pos*; e.g. hallucinations, delusions, conceptual disorganization), seven items assessing negative symptoms (*Neg*; e.g. blunted affect, passive/apathetic social avoidance) and sixteen items assessing global psychopathology (e.g. depression, anxiety, lack of insight, guilt). All items are scored between 1 (not present) and 7 (severe). A number of studies have demonstrated the reliability and validity of the PANSS. PANSS raters (RB, SH) were trained using a standardised approach and had achieved good inter-rater reliability (ICC = 0.874, P<0.001).

#### Beck Hopelessness Scale

The Hopelessness Scale (BHS[13]) was developed as a self-report measure regarding pessimism about the future. There are 20 true/false items and scores of 9 or above indicate a high risk of future suicide[14], [15]. It has a demonstrably high internal consistency and scores are associated with levels of depression[13].

#### Hospital Anxiety and Depression Scale

The hospital anxiety and depression scale (HADS; [16] is a 14 item self-report measure. Of these items, 7 assess depression, whilst the remaining 7 assess anxiety over the preceding week. Cronbach's alpha values for anxiety and depression scales for this sample are α = 0.86 and α = 0.83 respectively. Because these scales were highly correlated (r = .67, p<000) scores were added to derive a single dysphoria score for subsequent analyses (alpha = .90) (Using separate anxiety and depression scores did not appreciably alter any of the results).

#### Birchwood Insight Scale

Insight was measured with the Insight Scale (*IS*; [17], an 8-item self-report scale designed to be sensitive to change, and which captures three widely accepted dimensions of insight: perceived need for treatment, awareness of illness and relabeling of symptoms as pathological. Higher scores indicate greater levels of insight. In this study the scale had an alpha coefficient of 0.66.

### Brief Assessment of Cognition in Schizophrenia (BACS)

The Brief Assessment of Cognition in Schizophrenia (BACS; [18], [19] is a battery of neurocognitive tests that assesses domains of cognition found to be most impaired and most strongly correlated with outcome in patients with a diagnosis of schizophrenia. These include verbal memory, working memory, motor speed, verbal fluency, attention and speed of processing and executive function. The BACS has 6 subscales (list learning, digit sequencing task, token motor task, verbal fluency, Tower problem solving, and symbol coding), takes approximately 35 minutes to complete and has high levels of test re-test reliability on each of the subscales and with parallel forms for those subtests where they were available. In this study, the token motor task (in which participants are asked to pick up small tokens in both hands simultaneously and place them in a basket) was omitted after it was found to be poorly tolerated. Therefore rather than use the total BACS score the data were factor analysed and the single resultant factor used in susequent analyses. The BACS has an equivalent sensitivity to the detection of impairments as a standard, more extensive, neuropsychological test battery [19].

### Subjective Scale to Investigate Cognition in Schizophrenia (SSTICS)

The SSTICS [20] is a brief, 21 item self report scale examining subjective complaints about memory, attention, praxia and executive function in patients affected by schizophrenia. In the original study it exhibited good internal consistency (α = .86) and test-retest reliability (r_s_ = .82). In this study, the internal consistency (α) was.90 across the two groups. Specific areas of cognition covered are working memory, explicit memory (divided into episodic and semantic memory), attention, executive function and language. There are five subscales: explicit memory, episodic memory, semantic memory, working memory and attention. Stip et al. reported significant but weak associations between the memory subscales and objectively assessed mnemonic function. The 21 items take approximately six minutes to complete.

## Results

### Comparison of groups

Table 1 shows mean scores of the two groups on measures of mood, subjective and objective cognition. Significant differences were observed between the groups on all of the variables with the exception of the SSTICS working memory subscale. Pearson correlation coefficients revealed a modest and marginally significant correlation between total BACS and total SSTICS scores for the non-clinical comparison group (r = −.31, p = .05, n = 41) but no correlation for the patients (r = −.05, p = .62, n = 117). Although these correlations differed in terms of significance level, they did not differ significantly from each other (z = 1.44, ns).

**Table 1:** Between group differences in depression, anxiety, objective cognition and subjective cognition.

	Patients			Controls			
	Mean	SD	N	Mean	SD	N	p
HADS Depression	6.11	4.37	110	2.69	2.82	42	<0.001
HADS Anxiety	8.78	4.98	110	4.98	3.32	42	<0.001
BACS							
Verbal memory	30.77	11.18	112	48.80	8.64	45	<0.001
Letter fluency	20.99	7.89	112	31.04	9.42	45	<0.001
Digit sequencing	15.89	3.97	112	20.96	3.72	45	<0.001
Semantic fluency	19.64	6.38	112	27.04	5.66	45	<0.001
Symbol coding	39.23	13.55	111	60.98	11.01	44	<0.001
Tower of London	30.49	8.10	111	35.91	4.11	44	<0.001
SSTICS							
Memory total	16.77	7.47	112	13.29	7.46	42	0.011
Explicit memory	12.02	6.00	112	8.83	5.93	42	0.004
Episodic memory	8.96	4.66	112	6.83	4.94	42	0.014
Semantic memory	3.05	2.17	112	2.00	1.65	43	0.005
Working memory	4.75	2.04	112	4.51	1.94	43	ns
Attention	8.84	4.44	112	5.67	4.02	43	<0.001

It might be argued that insight should moderate the relationship between BACS and SSTICS, so that only patients with good insight will correctly judge their cognitive ability. To test for this possibility, a multiple regression was carried out using centred variables, with SSTICS total scores as the dependent variable, total insight and total BACS scores entered as predictors in the first stage, and then the interaction between the BACS and insight scores entered in the second stage. When the BACS and insight variables were entered, there was a significant model (F_[2,103]_ = 4.113, p<.02, adjusted r^2^ = .056) but only insight was retained as a significant predictor (**β** = .27, t = 2.86, p<.005). However, adding the interaction between insight and BACS scores in the second stage did not improve the model (F_change_ = .55, p = .46) and the effect of the interaction was nonsignificant.

### Predictors of subjective cognitive impairment in the patient group

Correlations between SSTICS and BACS subscales for the patient group are shown in Table 2 with those for the non-clinical comparison group summarized in Table 3. It can be seen that none of the SSTICS subscales were associated significantly with any of the BACS subscales in the clinical group. In addition the SSTICS total scores and single factor scores on the BACS (see below) were not associated (Pearson's r = .006; ns). One possibility was that level of education may have not only been related to BACS scores, but also the reporting of SCC. Table 4 provides a summary of correlations between the SSTICS, BACS factor scores and symptom variables. It can be seen that positive symptoms, insight and dysphoria exhibited significant correlations with the SSTICS such that better insight, worse positive symptoms and greater dysphoria were associated with poorer subjective cognitive functioning. It is noteworthy that dysphoria was associated significantly with insight and positive symptoms. Using the highly conservative Bonferroni method of correcting for multiple comparisons between the four clinical scales (PANSS positive, PANSS negative, dysphoria and insight) and the two cognitive measures (BACS and SSTICS) does not alter the overall picture (critical p = .00625).

**Table 2:** Correlations between STICSS and BACS subscale scores in the patient group.

SSTICS sub-scale		Digit sequencing Total	Semantic Fluency	Symbol Coding	Verbal Memory	Letter Fluency	Tower of London
Memory Total	Pearson's r	−.107	−.015	.099	−.094	−.010	.147
	p	.263	.879	.300	.325	.917	.124
	n	112	112	111	112	112	111
Explicit Memory	Pearson's r	−.128	−.013	.070	−.091	−.023	.149
	p	.179	.894	.463	.338	.813	.118
	n	112	112	111	112	112	111
Episodic Memory	Pearson's r	−.124	−.053	.022	−.099	−.060	.126
	p	.193	.582	.817	.301	.527	.187
	n	112	112	111	112	112	111
Semantic Memory	Pearson's r	−.088	.077	.146	−.041	.067	.146
	p	.356	.417	.125	.668	.484	.127
	n	112	112	111	112	112	111
Working Memory	Pearson's r	−.014	−.016	.157	−.075	.030	.098
	p	.880	.868	.099	.430	.753	.308
	n	112	112	111	112	112	111
Attention Total	Pearson's r	.043	−.043	.124	.067	.063	.136
	p	.653	.652	.193	.481	.510	.153
	n	112	112	111	112	112	111
Executive Function	Pearson's r	.043	.012	−.112	.002	.004	.075
	p	.653	.903	.241	.985	.964	.431
	n	112	112	111	112	112	111

** Correlation is significant at the 0.01 level (2-tailed).

* Correlation is significant at the 0.05 level (2-tailed).

**Table 3:** Correlations between STICSS and BACS subscale scores in the non-patient group.

SSTICS sub-scale		Digit sequencing Total	Semantic Fluency	Symbol Coding	Verbal Memory	Letter Fluency	Tower of London
Memory Total	Pearson's r	−.278	−.142	−.172	−.307*	−.211	−.275
	p	.074	.370	.281	.048	.181	.078
	n	42	42	41	42	42	42
Explicit Memory	Pearson's r	−.225	−.134	−.143	−.318*	−.201	−.267
	p	.153	.397	.372	.040	.201	.088
	n	42	42	41	42	42	42
Episodic Memory	Pearson's r	−.209	−.165	−.118	−.304*	−.170	−.272
	p	.184	.297	.464	.050	.281	.082
	n	42	42	41	42	42	42
Semantic Memory	Pearson's r	−.177	.010	−.160	−.228	−.210	−.143
	p	.257	.948	.313	.142	.177	.362
	n	43	43	42	43	43	43
Working Memory	Pearson's r	−.340*	−.189	−.234	−.227	−.214	−.224
	p	.026	.225	.135	.143	.168	.149
	n	43	43	42	43	43	43
Attention Total	Pearson's r	−.089	−.274	−.042	−.271	−.247	−.335*
	p	.570	.075	.789	.079	.110	.028
	n	43	43	42	43	43	43
Executive Function	Pearson's r	−.081	−.311*	−.067	−.238	−.030	−.068
	p	.606	.042	.674	.124	.846	.665
	n	43	43	42	43	43	43

** Correlation is significant at the 0.01 level (2-tailed).

* Correlation is significant at the 0.05 level (2-tailed).

**Table 4:** Correlations (Pearson) between BACS, SSTICS and clinical variables in the patient group.

		BACS factor score	CPZ daily equivalents	Duration of Psychosis (months)	PANSS Positive	PANSS Negative	Insight	Dysphoria (HADS)
Chlorpromazine daily equivalents	r	−.054						
	p	.613						
	n	90						
Psychosis Duration (months)	r	−.219*	.393^***^					
	p	.025	.000					
	n	105	88					
PANSS Positive	r	−.166	.085	.209*				
	p	.084	.417	.030				
	n	110	93	108				
PANSS Negative	r	−.525**	.230*	.033	.333**			
	p	.000	.028	.733	.000			
	n	109	92	107	114			
Insight	r	.063	.140	.172	.130	.004		
	p	.523	.186	.081	.173	.969		
	n	106	91	104	111	110		
Dysphoria (HADS)	r	−.007	.005	.058	.542**	.200*	.294**	
	p	.947	.966	.559	.000	.037	.002	
	n	108	90	103	110	109	106	
SSTICS	r	.006	−.095	.093	.321**	−.029	.210*	.594**
	p	.947	.367	.346	.001	.762	.029	.000
	n	110	92	105	112	111	108	110

** Correlation is significant at the 0.01 level (2-tailed).

* Correlation is significant at the 0.05 level (2-tailed).

Spearman correlations revealed that whilst the BACS factor was associated significantly with educational attainment (r = .37, p<.01) the SSTICS total scores were not (r = .03, ns). A further possible confound is that of diagnosis. Given the inclusion of participants with schizoaffective disorder and other psychoses the bivariate correlations between dysphoria and SSTICS total score was re-run with only those with a primary diagnosis of schizophrenia. The correlation remained significant (r = 0.66, p<.000, n = 72).

Linear regression was used to assess the relative value of relevant variables in predicting subjective cognition (SSTICS total scores). 13 participants were excluded from the analyses because of missing data on one or more scale. For the purpose of analysis, principle components analysis was used to derive a single factor score for the BACS (Eigenvalue = 2.991, % variance accounted for = 49.84, minimum loading = .58). Variables were entered in three stages: first BACS scores, second clinical variables (PANSS positive and negative and Birchwood Insight scores), and finally mood measures (HADS dysphoria and the Beck Hopelessness Scale scores). At each stage, the variables were entered simultaneously. Finally, the analyses were repeated with medication dose and duration of illness (log transformed) included at the first stage (this further reduced the sample size to 82 because data were missing for these variables for some of the participants.)

It can be seen from Table 5 that BACS scores did not significantly predict subjective cognitive functioning (p for initial model = .90). At the second stage, a significant model was generated (F[4,97] = 5.04, p<.001; adjusted R^2^ = .14) and greater insight and positive symptoms of psychosis predicted SSTICS scores. Adding the dysphoria variables improved the model (final F[6,95] = 10.78; adjusted R^2^ = .37, p<.001). At this stage, dysphoria was a significant predictor but the findings also suggested that negative symptoms were weakly although negatively associated with subjective cognitive function (less severe negative symptoms were associated with poorer subjective cognitive functioning).

**Table 5:** Multiple regression analysis 1.

Step		B	Std. Error	β
1	Constant	32.27	1.37	
	BACS Factor score	−.169	1.37	−.012
2	Constant	14.028	6.715	
	BACS Factor score	−.281	1.468	−.021
	PANSS Positive Total	.825	.249	.337**
	PANSS Negative Total	−.379	.328	−.132
	Insight	1.077	.445	.227*
3	Constant	21.031	5.870	
	BACS Factor score	−1.501	1.274	−.110
	PANSS Positive Total	.081	.246	.033
	PANSS Negative Total	−.586	.284	−.204**
	Insight	.396	.397	.084
	Hopelessness	−.069	.276	−.028
	Dysphoria (HADS)	1.002	.205	.630***

R^2^ step 1 = −.01; ΔR^2^ step 2 = .17; ΔR^2^ step 3 = .23. Final adjusted R2 = .

* p<0.05;

** P = 0.001;

*** p<0.001.

When the analyses were repeated with duration of illness and antipsychotic medication dose included, the results were essentially unchanged except that the effect for negative symptoms was removed (final F[8,73] = 7.27, p<.001; adjusted R2 = .38) and dysphoria remained the only significant predictor (see Table 6).

**Table 6:** Multiple regression analysis 2 (inclusion of duration of psychosis and medication level).

Step		B	Std. Error	β
1	Constant	27.448	7.805	
	BACS Factor score	.409	1.550	.030
	Duration of Psychosis	1.338	1.764	.095
	CPZ daily equivalents	−.007	.007	−.127
2	Constant	8.857	9.735	
	BACS Factor score	1.144	1.654	.084
	Duration of Psychosis	.054	1.678	.004
	CPZ daily equivalents	−.010	.007	−.178
	PANSS Positive Total	.710	.268	.310**
	PANSS Negative Total	.118	.362	.042
	Insight	1.258	.479	.284**
3	Constant	11.796	8.323	
	BACS Factor score	−.192	1.421	−.014
	Duration of Psychosis	1.025	1.434	.073
	CPZ daily equivalents	−.009	.006	−.154
	PANSS Positive Total	−.018	.261	−.008
	PANSS Negative Total	−.097	.315	−.035
	Insight	.599	.424	.135
	Hopelessness	−.211	.299	−.092
	Dysphoria (HADS)	.988	.206	.680***

R^2^ step 1 = −.02; ΔR^2^ step 2 = .18; ΔR^2^ step 3 = .24.

* P = 0.01; ***p<0.001.

## Discussion

### Summary of results and implications

The aim of the present study was to determine the relative importance of possible predictors of subjective cognitive complaints in schizophrenia and to replicate the finding that patients' subjective experiences of cognitive functioning fail to reflect objectively assessed cognitive performance[2], [3], [4], [5]. As in previous studies, SCC was not associated with cognitive test performance in patients with psychosis. However, in the non-clinical comparison group there was an association between cognitive function and SCC, indicating that healthy individuals are able to more accurately appraise their own mental functioning than patients. However, this association was relatively weak and the correlations between SCC and performance on the BACS in the clinical and non-clinical groups were not significantly different.

Some studies have shown that the association between cognitive performance and negative symptoms is mediated by defeatist beliefs [21], [22]. However, SCC as measured in the present study are unlikely to play this mediational role, as they failed to correlate with either negative symptoms or actual cognition. One possible explanation is that defeatist beliefs arise when cognitive failures that the patient is unaware of impair social functioning. However, it should be noted that in the first regression analysis (Table 5) it can be seen that negative symptoms are inversely related to SCC. In the final analysis this relationship disappeared once antipsychotic dose and duration of illness had been accounted for.

The present findings call in to question the use of the SSTICS as a means of evaluating cognition in those affected by psychosis. It has been argued that some everyday cognitive failures that are recognised by patients may be too subtle to be detected by neurocognitive tests [23]. However, other factors, included in the present study, appear to be important in determining patients' beliefs about their own cognitive functioning.

Consistent with a previous study of multiple factors [24], insight, anxiety and depression and positive symptoms were associated with subjective cognition. In the latter report the focus was on the role of insight; anxiety and depression were entered as covariates, leading to the relationship between insight and SCC being non-significant, but the authors did not highlight the implications of this finding. In fact, in the present study, anxiety and depression derived from the HADS were the only clear predictors of SCC once levels of medication, positive symptoms and negative symptoms had been accounted for. It is therefore likely that dysphoric symptoms account for patients' reports of cognitive problems.

It has been suggested that self-reports are affected in two ways. First, those affected by cognitive deficits may lack cognitive abilities to recognize deficits, whilst those whose abilities are unimpaired overestimate the scale of their problems. One possible explanation for the association between dysphoria and SCC is that people affected by anxiety and depression may be more likely to attribute their difficulties to poor memory or cognitive problems. This would be consistent with a substantial research literature that shows that depression is associated with a tendency to attribute negative experiences to internal causes [25]. Patients with depression may be excessively sensitive to normally occurring cognitive failures, taking the view that their ‘brain is broken’, especially if this message has been passed on to them by clinicians. It has been found that those experiencing psychosis, as well as those at risk of psychotic symptoms, exhibit less confidence in their cognitive abilities and are more self-conscious about them compared to controls [26]. These factors are a key aspect of certain anxiety disorders and depression where low ‘cognitive confidence’ plays a role in maintaining problems [27], [28].

In addition to the emotional aspects of SCC a further issue for investigation may be metacognition; that is the ability to recognize and manage one's own cognitive functioning. In this sense it should be related to insight. The present study suggests however, that insight has less predictive value for SCC once dysphoria is included in the equation. The same though may not be the case for broader areas of function. It is recognized for example that many patients under report problems with independent ‘real world’ functioning [29], but this is unlikely to be the result of depression or anxiety but is associated with insight[30]. In fact, depression and anxiety may result from realization of difficulties associated with psychosis [31] and are thus possibly inversely related to impairment of insight.

### Methodological limitations

There were certain limitations in the present study that should be considered. First, the non-clinical comparison group was not precisely matched to the patient group, especially in educational attainment. It was also was small (n = 45) compared with the clinical group. In addition there were some missing data, particularly concerning medication. This latter point is potentially important, because further questions may be raised about the roles of different categories of antipsychotic (e.g. first versus second generation treatments) as well as treatments for side effects.

Although the HADs is a widely used scale for determining levels of anxiety and depression, its use with participant groups affected by psychosis could be questioned. In relation to depression, scales have been developed focusing on signs and symptoms which are unlikely to be confused with antipsychotic medication side effects and negative symptoms[32], [33], [34]. This might have given a more rigorous specification of depression over and above these confounding aspects of psychotic disorders. The choice of the HADS was based upon the practical constraints of having to assess many patients and burdening them with a minimal interview length. It should be noted that one of the BACS subtests was dropped because of some participants' limited willingness to tolerate the testing. Future work I this area should try to better disentangle depression and anxiety from the effects of the psychotic disorders of concern and the side effects of their treatment.

A further potential measurement issue relates to the breadth and refinement of other symptom clusters. For example, one possibility in future work might be the evaluation of the role of other symptom factors derived from the PANSS[35]. For example what might be the role of the excitement and in particular cognitive/disorganized factors? This question could be answered by studies with larger participant samples with suitable levels of power allowing for the inclusion of more variables in regression analyses.

### Implications and future research

The major clinical implication is that clinicians should investigate and consider the role of depression and anxiety when confronted with patients with marked SCC. SCC in the absence of formal cognitive testing should not be considered evidence of impaired cognitive functioning. In addition, others have pointed out that reports from other informants can improve the accuracy of such judgments and where possible should be incorporated into interviews regarding cognitive performance [36], [37]. This is also the case in trials where interview based assessments of cognition are used as outcome variables [38].

Further more sophisticated methods might be used to examine the role of relevant variables in predicting SCC. For example Kim and Byun [6] found that, when medication doses were equated and patients divided by level of side effects, those reporting most side effects also reported higher levels of SCC. Thus there may be variables not associated with dysphoric symptoms or at least only partially so, that may explain SCC further, and which need to be considered in future studies. It would also be useful to investigate SCC longitudinally to determine, for example, whether they change over time with changing mood, and also whether they are more prominent at some stages of psychosis (e.g. acute episodes) than at others (e.g. during the prodromal period or in remission).
